# Future Directions for Integrative Objective Assessment of Eating Using Wearable Sensing Technology

**DOI:** 10.3389/fnut.2020.00080

**Published:** 2020-07-02

**Authors:** Andy Skinner, Zoi Toumpakari, Christopher Stone, Laura Johnson

**Affiliations:** ^1^School of Psychological Science, University of Bristol, Bristol, United Kingdom; ^2^MRC Integrative Epidemiology Unit, Bristol Medical School, University of Bristol, Bristol, United Kingdom; ^3^Centre for Exercise, Nutrition and Health Sciences, School for Policy Studies, University of Bristol, Bristol, United Kingdom

**Keywords:** objective, assessment, eating, wearable, technology

## Abstract

Established methods for nutritional assessment suffer from a number of important limitations. Diaries are burdensome to complete, food frequency questionnaires only capture average food intake, and both suffer from difficulties in self estimation of portion size and biases resulting from misreporting. Online and app versions of these methods have been developed, but issues with misreporting and portion size estimation remain. New methods utilizing passive data capture are required that address reporting bias, extend timescales for data collection, and transform what is possible for measuring habitual intakes. Digital and sensing technologies are enabling the development of innovative and transformative new methods in this area that will provide a better understanding of eating behavior and associations with health. In this article we describe how wrist-worn wearables, on-body cameras, and body-mounted biosensors can be used to capture data about when, what, and how much people eat and drink. We illustrate how these new techniques can be integrated to provide complete solutions for the passive, objective assessment of a wide range of traditional dietary factors, as well as novel measures of eating architecture, within person variation in intakes, and food/nutrient combinations within meals. We also discuss some of the challenges these new approaches will bring.

## Introduction

Non-communicable diseases now account for almost three quarters of global mortality, with cardiovascular disease (CVD) being the leading cause of death. Diet is responsible for more than half of CVD mortality worldwide (1). The proportion of diet-related deaths has remained relatively stable since 1990 suggesting interventions to improve food intakes have had limited success (1). A major issue in combatting diet-related disease is the way in which food intake and eating behavior are assessed. Accurate measurement of eating is key to monitoring the status quo and responses to individual or systems level interventions.

Recent years have seen a shift in nutritional science away from a focus on single nutrients such as saturated fats, toward a recognition that the complexity in patterns of food intake (e.g., combinations of foods and nutrients throughout the day), is more important in determining health (2–4). In addition to what we eat, we need to extend our understanding of eating architecture—the structure within which food and drinks are consumed. Factors such as the size, timing, and frequency of eating are increasingly recognized as independent determinants of health over and above what food is being eaten (5, 6). For example, skipping breakfast is consistently associated with higher body weight and poorer health outcomes (5, 7). Breakfast tends to be a small meal eaten in the morning made up of foods higher in fiber and micronutrients and it's not clear which of these features (meal size, timing, or food type), if any, are causing the benefits to health (8).

Traditional methods of dietary assessment, such as food diaries, 24-h recalls and food frequency questionnaires (FFQs), are self-reported and prone to substantial error and bias (9–11), which may distort diet and health associations (12). Misreporting is one widely recognized limitation of self-reported dietary assessment methods, with systematic under-reporting of energy intake identified in upto 70% of adults of adults in the UK National Diet and Nutrition Surveys (13, 14). Under-reporting occurs for a range of reasons including; difficulties estimating portion sizes for ingredients of complex meals, a desire to present one's diet positively (social desirability), and poor memory (11). People tend to under-report between-meal snacks, possibly because these snacks tend to be less socially desirable or because they are more sporadic, easily forgotten events (15).

Multi-day food diaries or 24-h recalls compare best with “gold standard” dietary biomarkers (16). But diaries or recalls are labor intensive for researchers to interpret and code, and burdensome for participants, which means data capture is limited to short time periods, (typically 3–7 days) and can take years to be available after collection (17). In addition, accurate memory is essential for 24 h recalls and even with prospective methods like food diaries, reactivity is a problem, where participants report accurately but eat less than usual because their eating is being recorded (10).

FFQs, although simpler and quicker to use, only capture average food intakes. Therefore, exposures increasingly acknowledged as important like the timing of eating (6), the way that foods are combined within a meal (18) and within person variation throughout the day or day to day (17) are unmeasured. With analyses of 4-day food diaries revealing that as much as 80% of food intake variation is within-person and only 20% variation between people (19), there are many untapped avenues for research into novel mechanisms relating diet to disease and identifying opportunities for interventions.

Online versions of “traditional” dietary assessment methods have been developed, but errors and biases remain. Validation studies of a range of online 24-h recall and food diary tools have shown the same problems as their paper-based equivalents; misreporting, portion size estimation, accurately matching foods consumed to foods in composition databases, and high participant burden (16, 20, 21). With the best methods currently available, on paper or online, a maximum of 80% of true intake can be captured and there are systematic differences in the 20% of food intake missing (10, 15).

There is a clear need to enhance dietary assessment methods to reduce error and bias, increase accuracy, and provide more detail on food intake over longer periods so that truly causal associations with health can be identified. A range of reviews and surveys have provided insights into the use of technology to advance dietary assessments (22–24). Recent reviews in particular have highlighted the potential for hybrid approaches that use multiple sensors and wearable devices to improve assessments (25–27). We offer an overview of the state of the art in the use of sensor and wearable technology for dietary assessment that covers both established and emerging methods, and which has a particular focus on passive methods—those that require little or ideally no effort from participants. We illustrate how integrating data from these methods and other sources could transform diet-related health research and behaviors.

## What we Eat

The most commonly used methods for objectively identifying food and portion sizes are image-based. The widespread adoption of smartphones (28) by most adults in high income countries means individuals always have a camera to hand as they go about their daily lives. Many smartphone apps exploring the use of food photography for dietary assessment have been developed and validated. Examples include the mobile food record (mFR) (29) and Remote Food Photography Method (RFPM) (30), where participants capture images of everything they eat over a defined time period by taking a photo before and after each meal. Initial problems with these methods included ensuring all meals were captured, and that photos captured all foods. There were also issues in identifying food items, both automatically and with manual coding systems. These apps were improved by adding customized reminders [drawing on ecological momentary assessment methods (31)], real-time monitoring of photos by researchers to encourage compliance, prompts to improve photo composition, and requests for supplementary information alongside photos. For example, users can confirm or correct tagged foods automatically identified in images (mFR) or add extra text or voice descriptions (RFPM).

The mFR and RFPM systems have been validated in adults using doubly labeled water (DLW) to assess the accuracy of energy intake estimated from several days of food photographs taken in free-living conditions. The mFR underestimated DLW measured energy expenditure by 19% (579 kcal/day), while the RFPM reported a mean underestimate of 3.7% (152 kcal/day), which is similar, if not slightly better, agreement than seen in self-reported methods (30). However, food photography currently has considerable researcher and participant burden because of the requirements for training, real-time monitoring, and provision of supplementary information. Crucially, participants still have to actively take photographs of everything they eat, and this may be affected by issues with memory and social desirability (32).

The introduction of wearable camera systems recording point of view images addresses some of these issues, by making the capturing of images of meals largely passive. Among the first wearable camera systems were those developed for life logging; recording images of events and activities throughout the day in order to aid recall for a variety of benefits (33, 34). Feasibility testing of one such device, SenseCam, which was worn around the neck and automatically took photographs approximately every 30 s, indicated it was promising in enhancing the accuracy of dietary assessment by identifying 41 food items across a range of food groups that were not recorded by self-report methods (35). However, wearing the device around the neck meant variations in body shape could alter the direction of the lens, so for some individuals the device did not record images of meals.

Another passive wearable camera system, e-Button, reduced the size of the device so that it could be worn attached to the chest (36). Chest mounting improved the ability of the device to capture images of meals. However, the system was a bespoke development, and the use of bespoke solutions produced in limited numbers brings challenges, including potentially high unit costs, limited availability of devices, and issues around ongoing technical support.

Recent studies in other research domains have used mass-market wearable cameras of a similar shape and size to e-Button. For example, studies of infant interactions with environments and parents have used pin-on camera devices that are widely available online as novelty “spy badges” (37, 38). These devices have many characteristics that make them ideal for capturing images of meals; their small form and light weight mean they can be easily worn on the body, and their low cost facilitates use at scale. However, these devices typically capture individual images or video sequences initiated by the user, so they lack the passive operation of devices like eButton that capture images automatically throughout the day.

If using camera devices that capture images throughout the day, the first major challenge is to identify which images contain food and drink. A camera taking photographs every 10 s and worn for 12 h a day for a week will capture nearly 30,000 images, of which perhaps only 5–10% contain eating events (39), so identifying food-related photographs is a non-trivial first step. Automatic detection of images containing food using artificial intelligence shows promise for photos taken in ideal conditions (achieving an accuracy of 98.7%) (39). However, photos taken with a wearable camera are uncontrolled and more susceptible to poor lighting and blurring, and the accuracy of identifying images that depict food ranges from 95% for eating a meal to 50% for snacks or drinks (39).

Once meal images have been identified, the next step is to code food content and portion size. Expert analysis of photographs by nutritionists is currently the most common method but requires trained staff, is time-consuming (typically months to return a dataset), and expensive (>$10 per image). Alternatively, automated food identification and portion size assessment, using machine learning (ML) methods, is complex and computationally intensive. The latest approaches using convolutional neural networks appear promising, with accuracy ranging from 0.92 to 0.98 and recall from 0.86 to 0.93 (40) when classifying images from a food image database (41) into 16 food groups. However, identification of individual food items remains limited (42). ML methods require large databases of annotated food photos to train their algorithms, which are time-consuming to create. With more than 50,000 foods in supermarkets (21) and product innovation changing the landscape constantly, considerable challenges remain for ML approaches.

Humans, on the other hand, have life-long experience visually analyzing food, and are excellent at food recognition. Crowdsourcing approaches, in which untrained groups of people perform a short, simple (usually Internet-based) task for a small fee, might therefore offer a rapid low-cost alternative to expensive experts while ML methods develop. Platemate is one dietary assessment app that employs this approach (43). It is an end-to-end system, incorporating all stages from photographic capture of meals through to crowd-based identification of all foods and their portion sizes and nutrient content. The system is complex, however, and by involving crowds of up to 20 people per photo it results in an average processing time of 90 min and cost of $5 per image. To be feasible for use in large-scale longitudinal studies or public health interventions, crowdsourcing of food data from photographs needs to be fast and low cost. We developed and piloted a novel system, FoodFinder (44), and found that small (*n* = 5) untrained crowds could rapidly classify foods and estimate meal weight in 3 min for £3.35 per photo. Crowds underestimated measured meal weight by 15% compared with 9% overestimation by an expert. A crowd's ability to identify foods correctly was highly specific (98%—foods not present in the photo were rarely reported) but less sensitive (64%—certain foods present were missed by the crowd). With further development crowdsourcing could be an important stepping-stone to the automated coding of meal images as ML methods mature. Crowdsourcing could also play an important role in this development, by creating annotated databases of meal photographs to facilitate training of ML algorithms.

In addition to image based methods for assessing meals, more recent developments in body-worn sensor technology have aimed to passively measure the consumption of specific nutrients. Small, tooth mounted sensors in which the properties of reflected radio frequency (RF) waves are modulated by the presence of certain chemicals in saliva can detect the consumption of salt and alcohol in real time (45). Similarly, tattoo like epidermal sensors that attach to, and stretch and flex with the skin can detect a variety of metabolites in an individual's perspiration that relate directly to their diet (46). For these devices, it is important that metabolites detected are specific to food intake, and not conflated with endogenous metabolites produced by the body as a result of eating.

To date these new oral and epidermal sensors have largely been tested in laboratory settings and are some way from becoming widely available. There are clearly compelling uses for these, for example accurate measurement of salt intake in patients with high blood pressure and sugar intakes in patients with diabetes, as well as enhancing food photography methods by providing non-visual nutritional composition information (e.g. sugar in tea or salt added in cooking). However, it is worth noting that these methods alone are not able to identify the food that contained these nutrients. For some dietary interests (e.g., changing dietary behaviors), food items need to be assessed rather than the nutrients they contain, and in these cases image-based methods for assessing meals will be required.

## When we Eat

To advance our understanding of the effects of diet, we require objective assessments of not just what we eat, but when we eat too. A variety of approaches for the passive detection of eating events have been proposed, including; acoustic methods using ear-mounted microphones to detect chewing (47), throat microphones to detect swallowing (48) and detection of jaw movements using different sensor types attached to the head or neck (49–52).

Although these methods are capable of detecting eating events passively, they need the individual to wear bespoke sensing devices attached around the head and neck, and when used on a daily basis this inevitably introduces a considerable level of device burden. To address this, one approach is to use sensors that are embedded in or attached to items that are already part of people's daily lives.

One method explored has been the use of sensors that are part of spectacles. Some approaches to this have used piezoelectric strain sensors on the arms of glasses that are attached to the side of the head to measure movements from the temporalis muscle when chewing (53, 54). High levels of performance have been reported with this approach, with one study reporting an area under the curve for chewing detection (in a combination of laboratory and free-living tests) of 0.97 (55). However, it does require the sensors be manually attached to the head every time the glasses are worn. Others have used electromyography, in which the electrical activity associated with temporalis muscle contraction is detected using sensors imbedded in the arms of 3D printed eyeglass frames (56). This also gives good performance, with recall and precision for chewing bout detection above 77% in free-living conditions. This approach does not need manual attachment of sensors, but it does require individually tailored glass frames to ensure sufficiently good contact of the built-in sensors with the head. More broadly, not everyone wears glasses, so there is also the issue of how these approaches would work for those who do not.

Another method is to use wrist-worn devices equipped with motion sensors to automatically detect eating events. Data from gyroscope and accelerometer motion sensors can be used to identify the signature hand gestures of certain modes of eating (57, 58). Early adopters of this approach strapped smartphones to the wrist (59). This functionality is now more conveniently available in the form of off-the-shelf activity monitors and smartwatches. These devices can be highly effective in detecting eating events, with recent reports of 90.1% precision and 88.7% recall (60). However, building recognition models that can generalize well in free-living conditions where unstructured eating activities occur alongside confounding activities can be challenging, and can result in reduced precision in detection (61).

Recent reviews concluded that smartwatches are of particular interest for eating as they represent an unobtrusive solution for both the tracking of eating behavior (62), and the delivery of targeted, context-sensitive recommendations promoting positive health outcomes (63), such as Just-in-Time interventions (64).

The latest ML techniques are enabling researchers to go beyond detection of eating events using wrist-worn wearables, to also measure within meal eating parameters such as eating speed. In a recent example, convolutional neural networks and long short-term memory ML methods were applied to data from the motion sensors in off-the-shelf smartwatches worn by 12 participants eating a variety of meal types in a restaurant (65). Sequences of bites were first detected, which were then classified into food intake cycles (starting from picking up food from the plate until wrist moves away from the mouth).

The ability to passively detect meal onset is an essential aspect of other healthcare systems too. One example is closed-loop artificial pancreas systems for the management of blood glucose in patients with type 1 diabetes. Such systems rely on detecting a rise in interstitial fluid glucose concentrations (a proxy for blood glucose) using continuous glucose monitors (CGM). Meal detection can be challenging as interstitial glucose rises well after a meal has begun, limiting the current use of CGM in real-time monitoring systems. However, meal detection models using CGM have developed from being purely computer-based simulations to now showing promise when fitted to real-world data. The mean delay in detecting the start of a meal has reduced from 45 to 25 min (66). CGM could therefore be another method for the passive, objective detection of meal timings in future, although further research, particularly in populations without diabetes, is required. Encouragingly pilot work in the US indicates that wearing a CGM for up to a week is as acceptable as wearing accelerometer-based sensors for 7 days (67). Furthermore, our own pilot work in the UK ALSPAC-G2 cohort demonstrated that using the latest CGM devices, which no longer require finger prick tests for calibration, improves uptake of 6 days of monitoring (68). This reflects a growing demand for non-invasive methods for CGM.

Photoplethysmography (PPG) is a technique that detects changes in levels of reflected light as a result of variation in properties of venous blood, and which is routinely included in off-the-shelf smartwatches and activity monitors for the measurement of heart rate. This same technique can also be used to non-invasively measure glucose levels, and the latest enhancements give measurement performance approaching that of reference blood glucose measurement devices (69). This opens the possibility that non-invasive CGM using commercially available smartwatches and activity monitors may be widely available in the near future, and theoretically devices of this kind could detect glucose patterns associated with meal start and end times. Once again though, the latency between start of meal and detection would need to be determined, and meal detection algorithms evaluated.

## Integrating Methods

The methods outlined above individually provide objective measurements of when, what and how much someone is eating. Integrating these methods offers the possibility of objectively capturing more complete and detailed pictures of dietary intake, while minimizing participant burden.

One previous proposal for an integrated system for objective dietary assessment involves combining smartwatch motion sensors with a camera built into the smartwatch (70). The motion sensors detect the start and stop of an eating event, and this triggers the camera to take an image of the meal for subsequent offline analysis. While this is a compact solution minimizing device burden, it does need the individual to direct the watch camera toward the meal to capture an image. More importantly, trends in smartwatch design have changed, and smartwatches typically no longer come equipped with built in cameras.

A more recent proposal again had a wrist-worn activity monitor to detect eating events, but this time combined with on-body sensors for detecting chewing and swallowing to capture more detailed information on bite count and bite rate within a meal (22). An interesting aspect of this system was the use of the individual's smartphone as the basis of a Wireless Body Area Network (WBAN) (71) to link up the activity monitor and different sensors. This enabled local communication between sensors via the smartphone, without the need to connect the sensors to a static wireless network or a cellular data connection.

**Figure 1:**
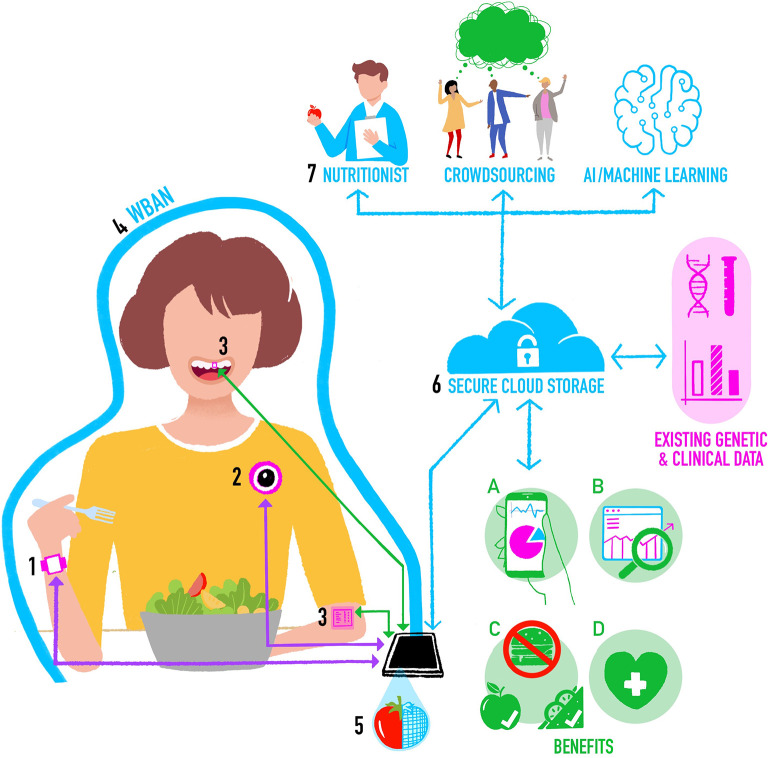
Integrating methods for objective assessment of diet using digital and sensing technologies.

In Figure 1 we propose a new architecture for an integrated system for objective assessment of diet. We draw on some elements of these previous proposals, but also incorporate new and future developments in wearable sensing technology for objective dietary assessment. The operation of the system can be conceptualized as follows:

1. The individual wears a smartwatch containing accelerometer and gyroscope motion sensors. Classification algorithms applied to the motion data in real time on the watch can detect the beginning and end of an eating event, the mode of eating, and provide “within meal” metrics such as speed of eating. In the future the smartwatch may also have PPG-based CGM, which provides additional data on meal timing and size.
2. The individual is also wearing a chest-mounted camera capturing images from their viewpoint. To keep battery consumption and data storage requirements low (minimizing device size and maximizing time between charges), the camera takes still images at short intervals (e.g., every 10 s) and stores them for a brief period (e.g., for 5 min). Images are then deleted unless the smartwatch detects the start of an eating event, in which case images before, during and after eating are stored as a complete visual record of the meal. Saving multiple photos maximizes the chances of capturing high quality images unaffected by temporary issues with lighting, camera angle, blur, etc.
3. On-body sensors including oral tooth-mounted sensors and epidermal tattoos could be added to provide more detailed nutritional assessments for monitoring of specific nutrients or calibrate estimates from other tools.
4. The individual's smartphone forms the basis of a WBAN around their body. Most devices (e.g., activity monitors, cameras) will communicate with the smartphone using a Bluetooth connection. Oral and epidermal sensing devices that do not currently have power supplies or data storage or transfer capabilities could use Near Field Communication (NFC) as a power source and to transfer data from the sensor to the smartphone.
5. Segmentation, food item recognition, and volumetric estimates of portion sizes are initially computed locally on the smartphone using data from sensors and images, and these may be used to support Just-in-Time type eating behavior change interventions.
6. The smartphone also provides a secure connection to a cloud-based central dietary profile for the individual. Data captured by sensors is processed on the smartphone and the processed data are regularly uploaded to the central profile, perhaps when the individual is at home and their smartphone connects to their home wireless network. Processed data can then, at the individual's discretion, be linked to other sources of their own health data, including omics, clinical, and imaging data. Raw data from sensors are not uploaded to reduce privacy concerns and data transfer requirements.
7. Depending on the needs of the particular scenario, and balancing speed, accuracy, and cost, data from the central profile may be sent for further analysis. For example, images of meals may be sent to a crowd-based application (44), or a dietician to refine food item identification and portion sizes (72).

The resultant cloud-based central dietary profile represents a detailed view of a person's food intake and eating behavior that will provide the following benefits:

- Summaries of the individual's data for their personal use.
- Dietary data that is stored and made available for future research on eating [for example prospective cohort studies like Children of the 90s (73)].
- Information that can be automatically analyzed within computer-based personalized nutrition behavior change interventions involving monitoring progress in achieving changes in diet-related goals [e.g., see (74)].
- Information that feeds into health professional consultations [e.g., enabling a dietician to get a better picture of an individual's overall intake and eating behavior so they can spend more time on behavior change techniques rather than having to assess diet as part of the appointment—for example see (75)].

## Discussion

In this article we briefly looked at how emerging digital and sensing technologies are enabling new objective assessments of dietary intake. These new methods have the potential to address many of the issues associated with current paper and online dietary assessment tools around bias, errors, misreporting, and high levels of participant or researcher burden. They do so by automating the detection and measurement of eating events, food items and portion sizes, and by providing detailed information on specific nutrients and within meal eating behaviors.

Image-based methods remain the most popular approach for objective assessment of food items and portion size. The use of on-body cameras to passively capture images of meals for subsequent processing has a number of advantages. As the individual does not have to manually initiate the capture, this helps mitigate issues such as the stigma of photographing their meals. The reliance on an individual's memory or willingness to self-report is also removed, therefore burden and bias are reduced. However, having to wear the camera device does represent a different burden, and there are issues around privacy, for example concerns from others that they may be inadvertently recorded. For nutritional assessment, image capture could be limited to eating occasions, so while concerns remain, they would hopefully be reduced.

In terms of camera devices, future developments should combine the passive operation and ease of use of a system like e-Button (36), with the low weight, size and cost, and broad availability of commercially available products. If such a device was of utility to multiple research domains (following the model of e-Button), and particularly if it had compelling mass market health or dietary use cases, demand could be sufficient for commercial production. Integration of such a device into other items already accepted for daily use (clothing, jewelery, etc.) could possibly increase acceptability further.

The emergence of sensors that attach directly to teeth or to the skin holds the promise of real time measurement of specific nutrients. These devices are at the proof of concept stage, and there are important considerations to address around durability, and how to power and read data from these devices. However, many of the mobile and wearable devices we currently use have capabilities that could possibly be adapted to work with these new sensors. For example, the near field communication wireless technology now included in most smartphones to make wireless card payments uses high frequency radio signals that could potentially be adapted to power and communicate with oral and dermal sensors (76).

In terms of detecting when people are eating, smart glasses could potentially detect the movement of, or electrical signals from the muscles used to chew, although there are the issues of sensor attachment and positioning, and how this approach would work for people who do not normally wear glasses. Wrist-worn wearables such as smartwatches have the ability to detect the signature hand movements unique to eating. Consumer demand for these devices continues to grow, with worldwide shipments predicted to exceed 300 million by 2023 (from under 30 million in 2014) (77). Smartwatches are worn by individuals as part of their daily routine so they do not represent additional device burden. In addition, such devices have the capability to run 3rd party applications providing the opportunity for delivering just-in-time behavior change interventions based on the eating behaviors detected. However, battery life continues to be an issue, with smartwatches typically needing to be charged daily. Continuous monitoring of eating behaviors will exacerbate this. Also, the detection of eating behaviors from motion data often use computationally intense machine learning algorithms (e.g., convolutional neural networks) that cannot currently be used on wearable devices to detect eating in real time. This may change in the future as the processing power and battery life of smartwatches and other wearables improve and more sophisticated classification algorithms can run on these devices.

In the current review, we have proposed an architecture for an integrated system for the objective assessment of diet. Integrating methods will enable researchers to build a more detailed and complete picture of an individual's diet, and to link this with a wide range of related health data (e.g., omics, clinical, imaging). Storing this information in a central location will enable healthcare professionals, researchers and other collaborators the individual wishes to interact with to have controlled access to their detailed dietary data. However, this raises a number of important questions. Should cloud-based storage be used and where this would be hosted? What format to use for the stored data to maximize utility across applications? What model should be used for making the data available, given the rise of new models in which individuals can monetise their own data? (78).

Another key issue for new methods will be that they need to be financially sustainable over time. For integrated systems, architectures are required that minimize the time and cost of maintaining operation of the system when one component (e.g., a sensor) changes. For example, systems arranged with central hubs to which each sensor/device connects and communicates reduce the impact of a change in one component compared with fully connected architectures in which each sensor/device communicates to many others.

For all of these new techniques for passive measurement of dietary intake, it will be important to understand if they introduce unexpected measurement errors and biases. New methods for estimating multiple sources of error in data captured using the latest technologies could help in this respect. These are able, for example, to separate out the effects of factors such as coverage (access to the technology), non-response and measurement error (79).

Finally, whether these methods, individually or integrated, become widely adopted will rest largely with the individuals that use them. Extensive feasibility testing will be required to explore which of these new methods people are happy to use, and which ones they are not.

## Author Contributions

AS, ZT, CS, and LJ contributed to conceptualizing and writing the article.

## Conflict of Interest

AS and CS are listed as inventors on patent applications 1601342.7 (UK) and PCT/GB2017/050110 (International), Method and Device for Detecting a Smoking Gesture. LJ has received funding from Danone Baby Nutrition, the Alpro foundation and Kellogg Europe for research unrelated to this review. The remaining author declares that the research was conducted in the absence of any commercial or financial relationships that could be construed as a potential conflict of interest.
